# Inhibition of lysosomal TRPML1 channel eliminates breast cancer stem cells by triggering ferroptosis

**DOI:** 10.1038/s41420-024-02026-y

**Published:** 2024-05-27

**Authors:** Chunhong Fan, Haotian Wu, Xin Du, Canjun Li, Wenping Zeng, Lili Qu, Chunlei Cang

**Affiliations:** 1 Key Laboratory of Immune Response and Immunotherapy, School of Basic Medical Sciences, Division of Life Sciences and Medicine, University of Science and Technology of China, Hefei, 230027 Anhui China; 2 Institute of Health and Medicine, Hefei Comprehensive National Science Center, Hefei, 230061 Anhui China; 3 Hefei National Research Center for Physical Sciences at the Microscale, University of Science and Technology of China, Hefei, 230027 Anhui China; 4 Department of Rheumatology and Immunology, The First Affiliated Hospital of USTC, Division of Life Sciences and Medicine, University of Science and Technology of China, Hefei, 230001 Anhui China

**Keywords:** Cancer stem cells, Cell death, Lysosomes, Transient receptor potential channels, Iron

## Abstract

Cancer stem cells (CSCs) are a sub-population of cells possessing high tumorigenic potential, which contribute to therapeutic resistance, metastasis and recurrence. Eradication of CSCs is widely recognized as a crucial factor in improving patient prognosis, yet the effective targeting of these cells remains a major challenge. Here, we show that the lysosomal cation channel TRPML1 represents a promising target for CSCs. TRPML1 is highly expressed in breast cancer cells and exhibits sensitivity to salinomycin, a drug known to selectively eliminate CSCs. Pharmacological inhibition and genetic depletion of TRPML1 promote ferroptosis in breast CSCs, reduce their stemness, and enhance the sensitivity of breast cancer cells to chemotherapy drug doxorubicin. The inhibition and knockout of TRPML1 also demonstrate significant suppression of tumor formation and growth in the mouse xenograft model. These findings suggest that targeting TRPML1 to eliminate CSCs may be an effective strategy for the treatment of breast cancer.

## Introduction

Breast cancer is the most prevalent form of cancer worldwide and remains one of the primary causes of mortality among women, with an increasing incidence rate each year [1]. Depending on the subtype and stage of breast cancer, patients usually undergo surgical intervention in conjunction with adjunctive therapies such as radiotherapy, chemotherapy, hormone therapy, and targeted therapy. Despite the efficacy of these treatments in eliminating a significant proportion of cancer cells, resistance to therapy and recurrence still pose frequent challenges. In recent decades, emerging evidence has revealed that a small subpopulation of cancer cells, known as cancer stem cells (CSCs), are the primary culprits in this major clinical challenge [2–4]. CSCs’ self-renewal, unlimited proliferation and differentiation potential maintain the vitality of cancer cells, while their migration ability plays a key role in tumor metastasis [5, 6]. The quiescence state endows CSCs with the ability to evade immune system surveillance and become insensitive to external physical and chemical cues [7, 8]. Moreover, compared with non-stem cancer cells, CSCs exhibit stronger metabolic plasticity that enables them to switch between glycolysis and oxidative phosphorylation in response to harsh external stimuli [9–11].

It is widely acknowledged that a tumor represents not only a cluster of cancer cells, but rather a complex entity consisting of heterogeneous cell populations, vascular networks, diverse secretory factors and extracellular matrix. Cancer cells constantly and dynamically interact with this microenvironment for adaption and survival [12, 13]. During this process, CSCs continuously remodel the microenvironment to create favorable conditions for tumor growth and invasion [2]. These properties of CSCs render complete elimination of cancer cells challenging. Therefore, identifying targets that can eradicate CSCs is urgently needed for effective breast cancer treatment.

To date, several targeted approaches for breast cancer stem cells (BCSCs) have yielded promising results. Salinomycin has garnered significant attention due to its potent and specific toxicity against BCSCs [14, 15]. Compared to non-cancerous cells, cancer cells require higher levels of iron for their continuous and rapid proliferation. Maintaining iron homeostasis in cancer cells, particularly CSCs, is crucial for tumor development [16, 17]. Salinomycin was found to bind to and sequester Fe^2+^ in lysosomes to block iron transport, leading to the production of lysosomal ROS and subsequent ferroptosis of CSCs [18, 19]. These findings suggest that disrupting lysosomal iron homeostasis may be an effective strategy for killing CSCs.

Transient receptor potential mucolipin 1 (TRPML1) is a crucial protein that regulates lysosomal iron homeostasis. It functions as a non-selective cation channel, facilitating the release of iron from lysosomes into the cytoplasm [20]. TRPML1 plays an essential role in maintaining lysosomal ion homeostasis and regulating autophagic flux. Defects in TRPML1 have been linked to Mucolipidosis type IV, a neurodegenerative lysosomal storage disorder [21, 22]. In recent years, emerging evidence has demonstrated the involvement of TRPML1 in various cancers, including breast cancer; however, its role in CSCs remains poorly understood.

In the present study, we investigated whether TRPML1 could serve as a viable target to interfere with iron homeostasis in CSCs. We find that salinomycin inhibits TRPML1, which may be one of the mechanisms by which salinomycin disrupts lysosomal iron homeostasis. Genetic depletion and pharmacological inhibition of TRPML1 promote ferroptosis in breast CSCs, reduce their stemness, and enhances the sensitivity of breast cancer cells to conventional chemotherapy drug doxorubicin (DOX). These findings suggest that targeting TRPML1 may represent a promising strategy for eradicating CSCs and developing effective antitumor therapies.

## Results

### Salinomycin inhibits TRPML1 in breast cancer cells and BCSCs

**Fig. 1:**
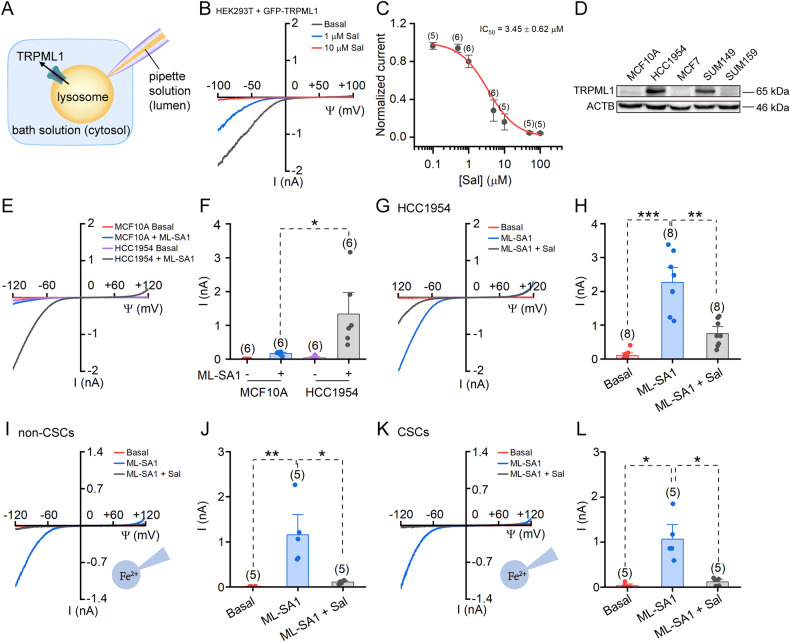
Salinomycin (Sal) blocks lysosomal cation channel TRPML1. **A** Diagram of the whole-lysosome patch-clamp recording. TRPML1-mediated cation flow from the lysosomal lumen into the cytosol, as shown by the arrow, is defined as an inward current. **B** Representative whole-lysosomal TRPML1 currents recorded in GFP-TRPML1 transfected HEK293T cells after bath application of 0, 1 and 10 μM salinomycin (Sal). The currents were elicited using ramp voltage protocols (−100 to +100 mV in 1 s, Vh = 0 mV). **C** Dose-dependent inhibition of TRPML1 current by Sal. **D** Western blot analysis of TRPML1 protein levels in MCF10A and breast cancer cells. **E** Representative whole-lysosomal TRPML1 currents recorded in MCF10A and HCC1954 cells, with or without bath application of 10 μM TRPML1 activator ML-SA1. The currents were elicited using ramp protocols (−120 to +120 mV in 2 s, every 10 s, Vh = 0 mV). **F** Current amplitudes measured at −120 mV in recordings shown in **E**. **G** Representative whole-lysosomal TRPML1 currents recorded in HCC1954 cells after bath application of 10 μM ML-SA1 alone or 10 μM ML-SA1 + 5 μM Sal. **H** Current amplitudes measured at −120 mV in recordings shown in **G**. Representative TRPML1 lysosomal currents recorded in non-CSCs (**I**) and CSCs (**K**) isolated from HCC1954 with 10 μM ML-SA1 alone or a combination of 10 μM ML-SA1 and 5 μM salinomycin (Sal) applied in bath solution. **J**, **L** Current amplitudes measured at −120 mV in recordings shown in **I** and **K**, respectively. Data are presented as the means ± SEM.

Given that TRPML1 is the primary iron-releasing channel on lysosomes, we initially investigated its potential involvement in salinomycin-induced iron accumulation within lysosomes. The TRPML1-mediated lysosomal currents were recorded using whole-lysosomal patch-clamp technique as previously described [20, 23] (Fig. 1A). In HEK293T cells exogenously expressing GFP-tagged TRPML1 (GFP-TRPML1), we observed a dose-dependent inhibition of TRPML1 currents by salinomycin (IC50 = 3.45 ± 0.62 μM; Fig. 1B, C). Next, we verified the impact of salinomycin on endogenously expressed TRPML1 in breast cancer cells. The expression level of TRPML1 protein was found to be significantly higher in the breast cancer cell line HCC1954 and SUM149 compared to the normal breast epithelial cells MCF10A (Fig. 1D). Consequently, a larger current activated by the TRPML1-specific agonist ML-SA1 was observed in HCC1954 than in MCF10A cells (Fig. 1E, F). The TRPML1 current in HCC1954 can also be inhibited by salinomycin (Fig. 1G, H). In addition, we sorted CSCs from HCC1954 cells with ALDEFLUOR assay by flow cytometry, and found that salinomycin also inhibited the ML-SA1 activated TRPML1 current in CSCs (Supplementary Fig. S1A, B).

Although lysosomes serve as crucial intracellular storage sites for ferrous iron (Fe^2+^), the concentration of Fe^2+^ in these vesicles is only in the micromolar range [24]. In order to better test whether salinomycin could regulate the permeability of TRPML1 to Fe^2+^, we replaced the 145 mM Na^+^ in the pipette solution with 105 mM Fe^2+^, thereby ensuring that Fe^2+^ was the sole cation in the pipette solution. Notably, salinomycin suppressed TRPML1-mediated Fe^2+^ currents in both CSCs and non-CSCs isolated form HCC1954 cells (Fig. 1I–L), as well as in TRPML1-overexpressed HEK293T cells (Supplementary Fig. S1C, D). These results suggest that salinomycin can modulate lysosomal iron homeostasis by acting on TRPML1 channels in breast cancer cells and CSCs, highlighting the potential of TRPML1 as an intervention site for breast cancer therapy.

### TRPML1 channels inhibitor ML-SI1 reduces the stemness of breast cancer cells

**Fig. 2:**
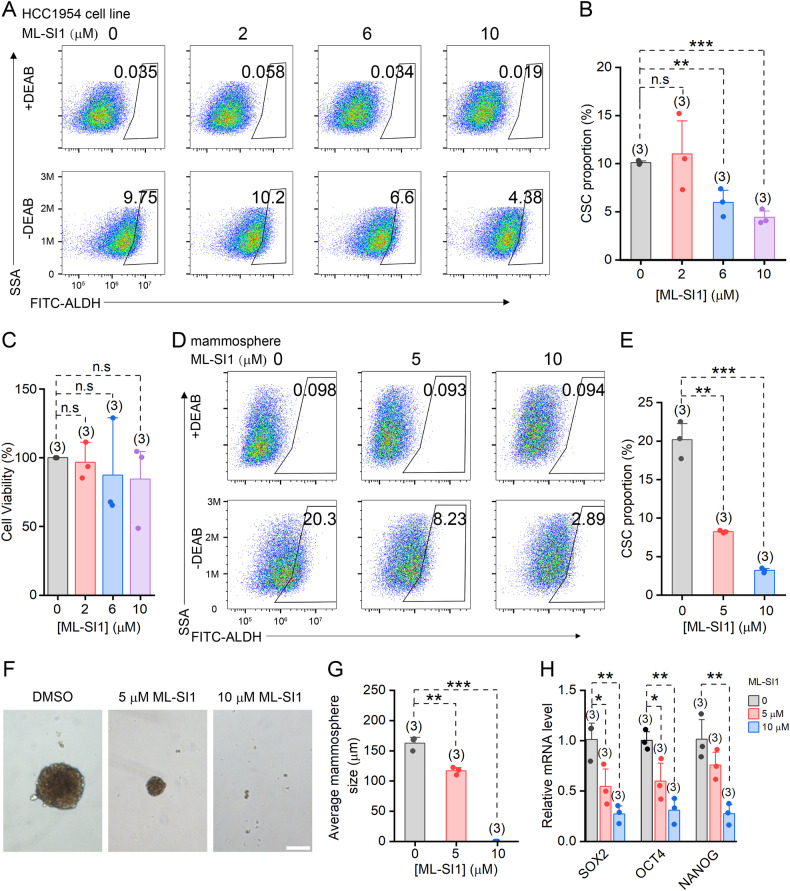
Inhibition of TRPML1 reduces the CSC fraction and stemness in HCC1954 cells. **A** Representative flow cytometry analysis of CSC proportion in HCC1954 cells following 48 h treatment of different concentrations of ML-SI1, using ALDEFLUOR assay. **B** Statistics of the CSC proportion in **A**. **C** Cell viability analysis measured by the CCK-8 assay. The percentage of viable cells relative to DMSO treatment after exposure to ML-SI1 for 48 h is indicated. **D** Representative flow cytometry analysis of CSC proportion in HCC1954 mammospheres following 48 h treatment of different concentrations of ML-SI1. **E** CSC proportion in HCC1954 mammospheres in **D**. **F** Effect of 5 μM and 10 μM ML-SI1 on mammosphere formation and proliferation. Scale bar = 100 μm. **G** The diameters of the mammospheres in **F**. **H** Relative mRNA levels of the stemness markers SOX2, OCT4 and NANOG in cultured mammospheres treated with 0, 5 or 10 μM ML-SI1. Data are presented as the means ± SEM.

Recently accumulated evidence has demonstrated the crucial role of iron metabolism in the survival of CSCs [25]. Salinomycin disrupts iron homeostasis by inhibiting TRPML1, which may represent a crucial mechanism underlying its selective toxicity against CSCs. To ascertain the significance of TRPML1 in targeting CSCs, we treated HCC1954 cells with different concentrations ML-SI1, a specific inhibitor of TRPML1, for 48 h. Subsequently, ALDEFLUOR assay was employed to identify and quantify CSCs using flow cytometry. It was noticed that the proportions of CSCs were significantly reduced by both 6 μM and 10 μM ML-SI1 (Fig. 2A, B). To further validate the efficacy of ML-SI1 in eradicating cancer stem cells, we also utilized CD44^+^/CD24^−^ as an additional marker for identifying these cells. The results demonstrated a robust reduction in the proportion of CD44^+^/CD24^−^ cells following treatment with ML-SI1 (Supplementary Fig. S2). The viability of HCC1954 cells remained unaffected by ML-SI1 unless the concentration was increased to 20 μM (Fig. 2C, and Supplementary Fig. S3). In addition, we isolated CSCs and non-CSCs from HCC1954 cells and treated them with ML-SI1 separately, and found that the viability of CSCs was significantly lower than that of non-CSCs after ML-SI1 treatment (Supplementary Fig. S4). These findings indicate that ML-SI1 exhibits enhanced sensitivity towards CSCs compared to non-CSCs, thereby elucidating its ability to reduce the proportion of CSCs.

To investigate the impact of TRPML1 inhibition on tumor formation, HCC1954 and SUM149 cells were cultured under non-adherent conditions for 7 days to generate de novo tumor-like structures known as mammospheres, which effectively enrich CSCs [26, 27]. Subsequently, the mammospheres were isolated by trypsinization and cultured for an additional 24 h prior to treatment with ML-SI1 for 48 h. Flow cytometry analysis revealed a significant reduction in CSC proportions upon exposure to ML-SI1 at concentrations of 5 μM and particularly at higher concentration (10 μM), indicating potent toxicity towards CSCs (Fig. 2D, E, and Supplementary Fig. S5). The decrease in CSCs is expected to result in a diminished capacity for secondary mammosphere formation. To validate this hypothesis, the isolated mammosphere cells were cultured for 8 days to form secondary mammospheres. Treatment with ML-SI1 in last 7 days significantly reduced the sizes of secondary mammospheres (Fig. 2F, G) and downregulated the mRNA levels of the pluripotent transcription factors SOX2, OCT4, and NANOG (Fig. 2H), thereby confirming the crucial role of TRPML1 in the survival and stemness of CSCs.

### ML-SI1 promotes ferroptosis in CSCs

**Fig. 3:**
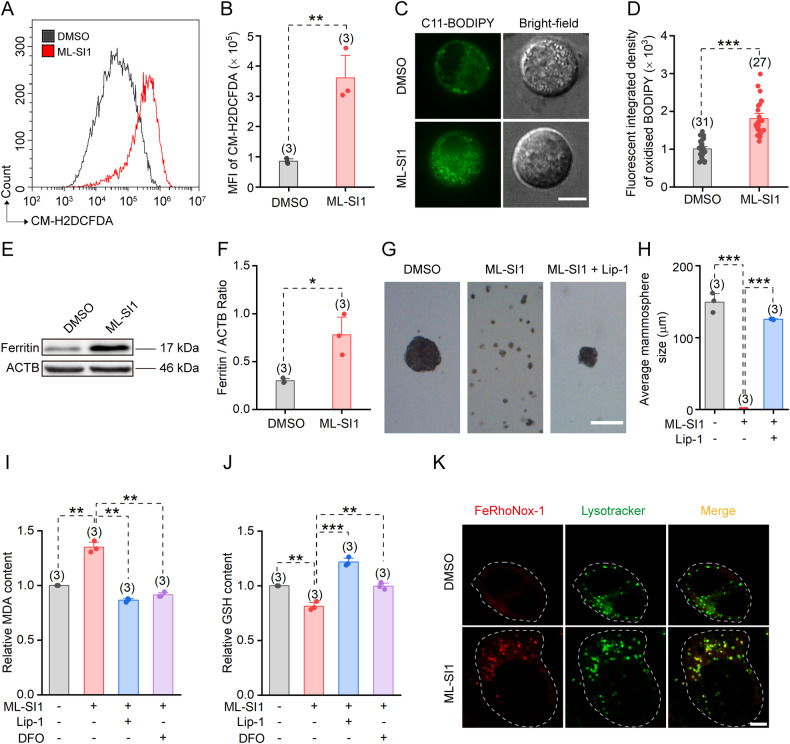
ML-SI1 promotes ferroptosis in CSCs. **A** ROS levels detected using the CM-H2DCFDA dye by flow cytometry in HCC1954 mammosphere cells incubated with DMSO or 10 μM ML-SI1 for 48 h. **B** Statistics of CM-H2DCFDA signals presented in **A**. **C** Confocal images showing lipid ROS visualized by C11-BODIPY in cells isolated from HCC1954 mammospheres and treated with 10 μM ML-SI1 for 48 h. Scale bar = 10 μm. **D** Statistics of C11-BODIPY signals presented in **C**. **E** Immunoblots of the Ferritin levels in HCC1954 mammospheres after 48 h treatment of DMSO or 10 μM ML-SI1. **F** Quantification of the relative Ferritin levels normalized to ACTB in **E**. **G** Images of HCC1954 mammospheres after treatment of 10 μM ML-SI1 alone or 10 μM ML-SI1 + 1 μM Liproxstatin-1 (Lip-1) for 7 days. Scale bar = 200 μm. **H** The diameters of the mammospheres in **G**. The relative MDA (**I**) and GSH (**J**) contents normalized to that of the control vehicle group in HCC1954 cells treated with 10 μM ML-SI1, 10 μM ML-SI1 + 1 μM Lip-1 or 10 μM ML-SI1 + 20 μM DFO as indicated. **K** Co-localization of FeRhoNox-1 with Lysotracker Green in HCC1954 cells pre-incubated with DMSO or 10 μM ML-SI1 for 48 h. Scale bar = 10 μm. Data are presented as the means ± SEM.

Dysregulation of lysosomal iron homeostasis results in the induction of ferroptosis, characterized by excessive generation of intracellular ROS and lipid peroxidation, also known as lipid ROS [28]. Inhibition of TRPML1 is likely to disrupt lysosomal iron homeostasis and induce ferroptosis in CSCs. To test this hypothesis, we treated CSC-enriched mammospheres with ML-SI1 and subsequently assessed the alteration in intracellular ROS levels using CM-H2DCFDA dye for general ROS detection and BODIPY 581/591 C11 dye specifically for lipid ROS visualization. The results showed that inhibiting TRPML1 caused a significant increase in the levels of ROS and lipid ROS in mammosphere cells, indicating the induction of ferroptosis (Fig. 3A–D).

Ferritin is the primary protein complex responsible for storing iron ions, and its degradation, specifically through a unique process known as ferritinophagy, results in the release of iron and subsequent oxidative damage [29, 30]. Increased ferritin expression is generally thought to limit ferroptosis [31]. However, our western blotting analysis revealed an upregulation of ferritin levels in ML-SI1-treated mammosphere cells (Fig. 3E, F). This observation aligns with previous reports indicating that promoting ferroptosis leads to an elevation in the level of ferritin heavy chain (FTH), which may represent a compensatory response by cells to a ferroptotic stimulus [30, 32].

Importantly, the inhibitory effect of ML-SI1 on the growth of the HCC1954 mammospheres could be reversed by the ferroptosis inhibitor Liproxstatin-1 (Lip-1) (Fig. 3G, H). Reduction in intracellular glutathione (GSH) levels and elevation in malondialdehyde (MDA) levels are characteristic features of ferroptosis [30]. Following a 48-h application of 10 μM ML-SI1, we observed increased MDA levels and decreased GSH levels in HCC1954 cells. Notably, these effects were significantly alleviated by Lip-1 and the iron-chelator deferoxamine (DFO) (Fig. 3I, J). We also measured Fe^2+^ storage in lysosomes using a cell-permeable Fe^2+^ probe FeRhoNox-1, which can generate an irreversible orange (red) fluorescent product once reacted with Fe^2+^. Our results revealed significant lysosomal accumulation of Fe^2+^ in ML-SI1 treated cells, which is likely to be the underlying cause for the induction of ferroptosis (Fig. 3K). Collectively, our findings demonstrate that inhibition of TRPML1 induces ferroptosis in BCSCs.

### ML-SI1 inhibits the migration of HCC1954 cells

**Fig. 4:**
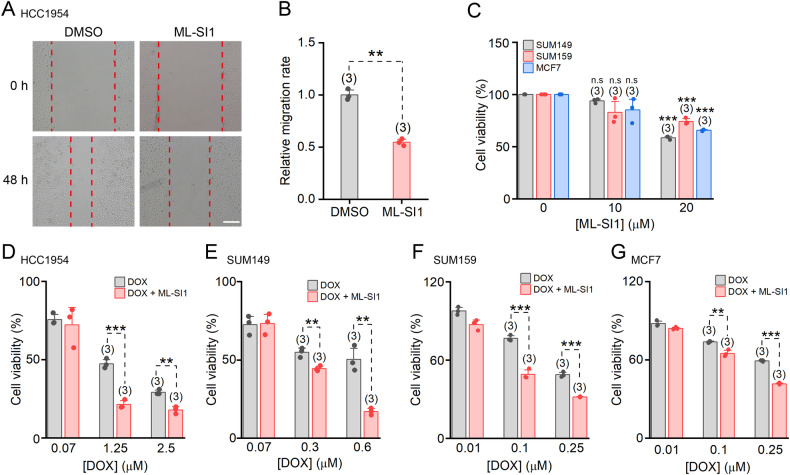
Effects of ML-SI1 on cell migration, drug sensitivity and ferroptosis in breast cancer cells. **A** Representative wound healing images of HCC1954 cells before and after incubation with DMSO or 10 μM ML-SI1 for 48 h. The wound was created by a straight-line scratch across the cell monolayer with a 1 mL pipette tip. The red dashed lines indicate wound edges. Scale bar = 400 μm. **B** Relative migration rates in **A**. **C** Cell viability assessed with a CCK-8 kit in SUM149, SUM159, and MCF cells treated with 0 μM, 10 μM, and 20 μM ML1-SI1. Viability of HCC1954 (**D**), SUM149 (**E**), SUM159 (**F**), and MCF7 (**G**) cells after 48 h treatment with DOX or DOX + 10 μM ML-SI1 as indicated. Data are presented as the means ± SEM.

CSCs play a pivotal role in facilitating the migration of cancer cells. To evaluate the impact of ML-SI1 on HCC1954 cell migration, we employed a wound healing assay. Specifically, HCC1954 cells were seeded onto 6-well plates and subjected to straight scratches prior to treatment with DMSO or ML-SI1 for 48 h. Notably, inhibition of TRPML1 remarkably impeded breast cancer cell migration ability (Fig. 4A, B).

### ML-SI1 augments drug sensitivity in breast cancer cells

Doxorubicin (DOX) is a prominent chemotherapeutic agent utilized in the treatment of breast cancer. The sensitivity of HCC1954 and SUM149 cells to DOX was enhanced upon treatment with 10 μM ML-SI1, a concentration that proved insufficient to affect cell viability (Figs. 2C and 4C–E). Additionally, we observed that ML-SI1 also augmented the cytotoxic effect of DOX in SUM159 and MCF7 cells (Fig. 4F, G), which express low levels of TRPML1 (Fig. 1D). However, this enhancement was less pronounced compared to that observed in HCC1954 and SUM149 cells. Overall, our findings suggest that inhibition of TRPML1 may enhance the efficacy of DOX therapy.

### TRPML1 down-regulation enhances the efficacy of salinomycin against CSCs

Salinomycin has strong toxicity [33, 34], which greatly limits its clinical application. Although salinomycin can inhibit TRPML1, higher concentrations are necessary to achieve optimal inhibitory effects (Fig. 1C), and these elevated concentrations often result in increased toxicity. We found that combining a lower concentration of salinomycin (1 μM) with ML-SI1 more effectively suppresses the current of TRPML1 in HEK293T and HCC1954 cells overexpressing GFP-TRPML1 (Supplementary Fig. S6). This suggests that targeting TRPML1 holds promise for enhancing the efficacy of salinomycin against CSCs.

**Fig. 5:**
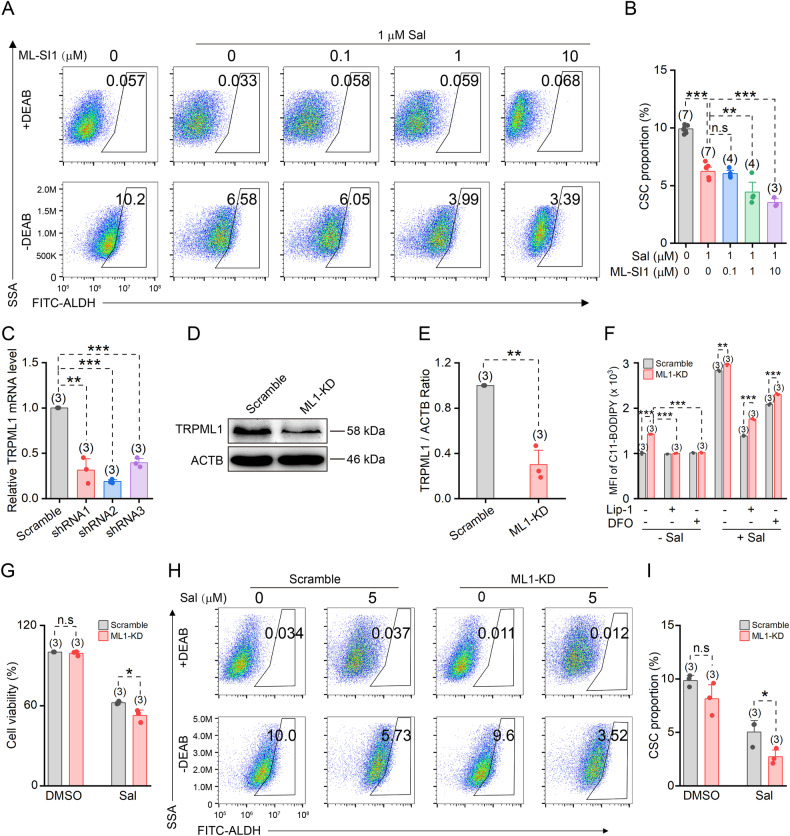
TRPML1 deficiency affects the drug sensitivity of CSCs to salinomycin. **A** Representative CSCs proportions assessed by flow cytometry using ALDEFLUOR assay in HCC1954 cells after 48 h treatment of 1 μM Sal and different concentrations of ML-SI1. **B** Statistics of the CSC proportions in **A**. **C** Relative mRNA levels of TRPML1 in scramble control or TRPML1 knockdown (ML1-KD) HCC1954 cells. **D** Western blot analysis of the TRPML1 levels in scramble control and ML1-KD cells. **E** Quantification of the TRPML1 levels normalized to ACTB in **D**. **F** Mean C11-BODIPY fluorescence intensity in scramble and ML1-KD cells. Cells were pre-incubated with either 1 μM Lip-1 or 20 μM DFO for 48 h in the presence or absence of 5 μM Sal before lipid ROS levels measurement. **G** Cell viability of scramble and ML1-KD HCC1954 cells pretreated with vehicle or 5 μM Sal for 48 h. **H** Representative CSC proportions in scramble and ML1-KD HCC1954 cells treated with or without 5 μM Sal for 48 h. **I** Quantification of the CSC proportions in **H**. Data are presented as the means ± SEM.

We subsequently treated HCC1954 cells with either salinomycin alone or a combination of salinomycin and ML-SI1 for 48 h, followed by the assessment of CSC proportions with ALDEFLUOR assay. Flow cytometry analysis revealed that both 1 μM and 10 μM ML-SI1 enhances the inhibitory effect of salinomycin on CSCs (Fig. 5A, B). Our previous findings have demonstrated that a concentration of 2 μM ML-SI1 was insufficient to effectively inhibit CSCs (Fig. 2A, B). Hence, the results obtained at a concentration of 1 μM indicate a potential synergistic effect between ML-SI1 and salinomycin.

Next, we used short hairpin RNA (shRNA) to knock down TRPML1 in HCC1954 cells (Fig. 5C). Among the three different shRNAs tested, shRNA2 exhibited the most pronounced effect. Thus, the resulting cells from shRNA2 treatment were designated as ML1-KD and used for subsequent experiments. Western blot analysis further confirmed a reduction in TRPML1 protein levels in ML1-KD cells (Fig. 5D, E). Interestingly, an elevation in lipid ROS was observed in ML1-KD cells, which could be attenuated by Lip-1 and DFO treatment (Fig. 5F). Notably, significantly higher lipid ROS levels were detected in HCC1954 cells treated with salinomycin. Although both Lip-1 and DFO effectively reduced the sharp rise of lipid ROS caused by salinomycin, the level of lipid ROS remained significantly higher in ML1-KD cells due to simultaneous suppression of TRPML1 protein expression level and activity (Fig. 5F). Furthermore, treatment of scramble-control and ML1-KD cells with salinomycin revealed a more pronounced reduction in the cell viability and proportion of CSCs specifically within the ML1-KD cells, suggesting that downregulation of TRPML1 enhances the efficacy of salinomycin against CSCs (Fig. 5G–I).

### Knockout of TRPML1 promotes ferroptosis in CSCs

**Fig. 6:**
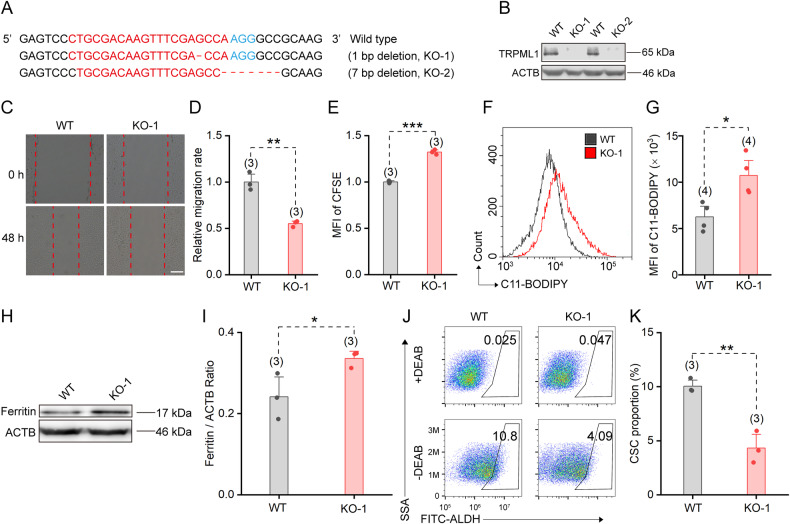
Knockout of TRPML1 promotes cellular ferroptosis and slows cell proliferation. **A** Schematic diagram showing knockout of TRPML1 in HCC1954 cells. **B** Western blot verification of TRPML1 knockout. **C** Representative wound healing images of WT and TRPML1-KO cells captured at 48 h after scratch. Scale bar = 400 μm. **D** Relative cell migration rates in WT and TRPML1-KO cells in **C**. **E** Normalized MFI of CFSE in WT and TRPML1-KO cells. **F** Lipid ROS levels were assessed by flow cytometry using lipid ROS sensor C11-BODIPY in WT and TRPML1-KO HCC1954 cells. **G** Quantitative analysis of lipid ROS levels detected in **F**. **H** Immunoblots of the Ferritin levels in WT and TRPML1-KO HCC1954 cells. **I** Quantification of the relative Ferritin levels normalized to ACTB in **H**. **J** Representative CSC proportions determination in WT and TRPML1-KO cells using ALDEFLUOR assay assessed by FACS. **K** Statistics of the CSC proportions in **J**. Data are presented as the means ± SEM.

To further validate the significance of TRPML1 in eradicating CSCs, we employed CRISPR-Cas9 technology to generate TRPML1 knockout HCC1954 cells. A single-guide RNA (sgRNA) was designed to target the second exon of TRPML1 coding gene. Subsequently, we successfully obtained two knockout cell lines harboring 1-bp or 7-bp deletions, respectively (Fig. 6A). The knockout of TRPML1 was further verified by western blot (Fig. 6B). One of the KO lines (KO-1) was used for subsequent in vitro experiments. The wound‑healing assay revealed an inhibition of cell migration ability in TRPML1 KO cells (Fig. 6C, D). Moreover, the proliferation rate of cells was markedly reduced in knockout cells, exhibiting an inverse correlation with the mean fluorescence intensity (MFI) of CFSE (Fig. 6E). Additionally, we observed an increase in the lipid ROS level in KO cells (Fig. 6F, G), accompanied by elevated levels of ferritin (Fig. 6H, I). As expected, the proportions of CSC were also significantly decreased in TRPML1-KO HCC1954 cells (Fig. 6J, K) and KO mammospheres (Supplementary Fig. S7). These results suggest that deficiency in TRPML1 retards cell migration, induces ferroptosis and decreases the proportion of CSC.

### ML-SI1 and knockout of TRPML1 inhibit xenograft tumor formation and growth

**Fig. 7:**
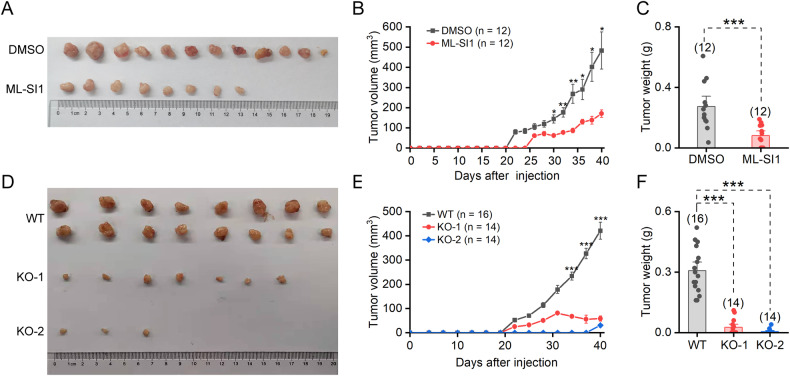
Effects of ML-SI1 and TRPML1-KO on tumor growth in breast tumor xenografts. **A** Tumors isolated from xenograft model mice 40 days after transplantation. The cells used for transplantation were isolated from secondary mammospheres treated with either DMSO or 20 μM ML-SI1 for 3 days. **B** The growth curves of the tumors in **A**. **C** Tumor weights in **A**. **D** Tumors isolated from xenograft model mice injected with WT or TRPML1-KO mammosphere cells 40 days after transplantation. **E** The growth curves of the tumors in **D**. **F** Tumor weights in **D**. Data are presented as the means ± SEM.

To evaluate the impact of TRPML1 inhibition on tumor growth in vivo, we employed a breast cancer xenograft tumor mouse model. Given that CSCs are considered crucial for tumor initiation and progression, injection of CSCs could more readily induces tumor formation in animal hosts. We pre-incubated the cultured mammospheres with DMSO or 20 μM ML-SI1 for 3 days and subsequently transplanted dispersed mammosphere cells into the mammary fat pad of nude mice. The tumor volume and tumor weight were significantly reduced in ML-SI1 treatment group (Fig. 7A–C). In another in vivo experiment, HCC1954 cells were directly injected into the mammary fat pad of nude mice, followed by intraperitoneal injections of ML-SI1 every other day. After a period of 50 days, xenograft tumors were excised and imaged. Continuous administration of 30 mg/kg ML-SI1 resulted in decreased tumor sizes (Supplementary Fig. S8A, B), while not affecting the body weight of the mice (Supplementary Fig. S8C).

Additionally, we injected WT or TRPML1-KO mammosphere cells (Fig. 7D–F) or HCC1954 cells (Supplementary Fig. S8D–F) into nude mice and monitored the tumor growth twice a week. The tumors in WT group mice exhibited significantly faster growth compared to those transplanted with KO cells. Accordingly, the tumor weight acquired was significantly reduced in KO group. These results collectively suggest that both pharmacological inhibition and genetic depletion of TRPML1 can effectively inhibit tumor initiation and growth.

## Discussion

Targeting CSCs is a significant challenge in cancer treatment due to their resistance to existing anti-tumor therapies [35]. Therefore, it is imperative to identify new targets and develop innovative strategies for the elimination of CSCs. In this study, we demonstrate that pharmacological inhibition and genetic depletion of the lysosomal cation channel TRPML1 induce ferroptosis-mediated death of CSCs, resulting in reduced stem cell proportion, enhanced chemosensitivity of tumor cells, and inhibited tumor growth. These findings suggest that targeting TRPML1 could be an important strategy for intervening CSCs.

TRPML1 was previously known to be associated with mucolipidosis type IV, a lysosomal storage disorder [22]. However, in recent years, its role in cancer has also garnered increased attention. TRPML1 exhibits high expression in various cancer types, including melanoma [36], pancreatic ductal carcinoma [37], triple negative breast cancer [38], and cancer cells bearing oncogenic *HRAS* mutations [39]. Our study also revealed elevated levels of TRPML1 in the breast cancer cell line HCC1954 and SUM149 compared to non-tumorigenic breast epithelial cell MCF10A (Fig. 1D). The upregulation of TRPML1 is positively correlated with tumor proliferation, migration, invasion, and poor prognosis [37–40]. Recent studies have revealed that loss of TRPML1 function impedes the migration of various cancer cells in vitro and reduces cancer dissemination in vivo [41]. Similarly, our study also found that pharmacological inhibition or genetic depletion can suppress the migration of HCC1954 cells, indicating that TRPML1 may serve as a promising target for anti-tumor therapy [37–39, 41]. However, to our knowledge, the role of TRPML1 in targeting CSCs has yet to be reported.

TRPML1 is a non-selective cation channel located on lysosomes, which are intracellular stores for metal ions such as calcium, zinc, and iron. These metal ions participate in various lysosomal functions and cellular activities [42]. By facilitating the release of these ions from lysosomes, TRPML1 plays a crucial role in the regulation of these cellular events. For instance, TRPML1-mediated lysosomal Zn^2+^ release induces mitochondrial dysfunction and necrotic cell death in metastatic melanoma cells [43], while lysosomal Ca^2+^ release through TRPML1 is implicated in multiple lysosomal-related processes such as lysosomal trafficking, autophagy, and organelle interactions [44]. Moreover, TRPML1-mediated lysosomal iron release is important for cellular iron metabolism [20].

Iron is an essential trace element, but excessive amounts can be toxic, thus necessitating tight regulation of iron metabolism. Iron is predominantly stored in its unreactive Fe^3+^ form inside the shell of ferritin at the cellular and organismal levels. Degradation of ferritin in lysosomes releases iron in the form of Fe^2+^, which subsequently catalyzes lipid peroxidation to an excessive extent and triggers ferroptotic cell death [45, 46]. Ferroptosis has gained significant attention in recent years due to its potential in cancer therapy [47]. CSCs have a higher demand for iron metabolism than non-CSC cells [48], making interfering with iron ion transport an effective strategy for targeting and killing CSCs. Previous studies have suggested that the divalent metal transporter (DMT1) is an endolysosomal iron transporter responsible for transporting free Fe^2+^ to the cytoplasm, and inhibition of DMT1 can enhance the mortality rate of CSCs [49]. Similar to DMT1, the endolysosomal ion channel TRPML1 also mediates the release of Fe^2+^ from lysosome to the cytosol, and its roles in regulating ferroptosis have also been revealed in recent studies [50, 51]. In this study, we also found that the TRPML1 inhibitor ML-SI1 could induce ferroptosis in CSCs and decrease their proportion in breast cancer cells. Although ion channels generally exhibit more efficient transmembrane ion transport compared to transporters, DMT1 may exert broader effects on iron homeostasis and ferroptosis than TRPML1. Firstly, TRPML1 is exclusively localized within the endolysosomes, whereas DMT1 is also found in the plasma membrane and mitochondrial membrane [52]. Secondly, the activity of TRPML1 is tightly regulated by factors such as endolysosomal membrane potential, lipid composition, mTOR signaling pathway. However, direct comparative studies investigating their respective roles in ferroptosis are currently lacking. Moreover, combined intervention targeting both iron regulators might potentially yield a stronger effect, which also needs further verification.

Breast malignant tumors exhibit high expression of stemness-related genes, including SOX2, OCT4, and NANOG in CSCs. Inhibiting the expression of these genes has been shown to suppress tumor growth [53–55]. Our study demonstrates that treatment with ML-SI1 reduces mRNA levels of SOX2, OCT4, and NANOG in HCC1954 mammospheres, providing further evidence for the potential of TRPML1 inhibition as a strategy to target CSCs.

In summary, our findings demonstrate the effectiveness of targeted inhibition of TRPML1 in eradicating CSCs, thereby providing a novel avenue for cancer therapy. Further investigations into the specific interference of TRPML1 in tumors or CSCs without compromising lysosomal homeostasis in normal cells, alongside the development of potent and selective TRPML1 inhibitors, hold great promise for advancing TRPML1-based approaches in cancer treatment.

## Materials and methods

### Animals

The 4–6-week-old female BALB/c nude mice were obtained from Shanghai SLAC Laboratory Animal Co. Ltd. The animal study was approved by the Animal Care and Use Committee of the University of Science and Technology of China. The mice were housed at a constant temperature (23 °C ± 1 °C) at a density of no more than 5 per cage. They were fed and watered ad libitum under specific pathogen-free conditions with a light/dark cycle set to 12 h. The sample sizes for animal experiments were determined based on established field standards and prior experience. Investigators are not blind in collecting data and analyzing results for animal experiments.

### Cell culture

The MCF10A, MCF7, SUM149 and SUM159 cell lines were generously provided by Dr. Tao Zhu (University of Science and Technology of China). The HCC1954 human breast cancer cell line and the HEK293T cell line were gifted by Dr. Dan Liu (University of Science and Technology of China). MCF10A cells were cultured in a Mammary Epithelial Cell Basal Medium (MEBM) (Lonza). SUM149 and SUM159 cells were cultured in Ham’s F12 medium (Gibco) supplemented with 10% FBS (Biological Industries), 4 μg/mL gentamycin (Aladdin), 1 μg/mL hydrocortisone (Aladdin), 5 μg/mL insulin (Aladdin) and 1% penicillin/streptomycin (Biosharp). HCC1954 cells were cultured in RPMI-1640 medium (Gibco) supplemented with 10% FBS (Biological Industries) and 1% penicillin/streptomycin (Biosharp). HEK293T and MCF7 cells were cultured in DMEM (Gibco) supplemented with 10% FBS, 1% GlutaMAX (Gibco), and 1% penicillin/streptomycin. All cultures were incubated at 37 °C under a humidified atmosphere containing 5% CO_2_. All cell lines were cultured for no more than 2 months before experiments and were confirmed to be free of mycoplasma contamination. The cells used for the patch-clamp recordings were seeded onto poly-L-lysine-coated circular coverslips 12 h prior to the experiment.

### cDNA constructs and transfection

The DNA constructs encoding human EGFP-tagged TRPML1 were a gift from Dr. Dejian Ren (University of Pennsylvania). DNA transfection was performed with PolyJet (SignaGen Laboratories) in accordance with the manufacturer’s instructions.

### TRPML1 knockdown in HCC1954 cell line

The knockdown of TRPML1 in HCC1954 cells was achieved through lentivirus-mediated delivery of shRNA. The shRNA constructs were generously provided by Dr. Ge Shan from the University of Science and Technology of China. The shRNA sequences for human TRPML1 (shRNA1, 5’-CCGGCGCCGTCGTCTCAAATACTTTCTCGAGAAAGTATTTGAGACGACGGCGTTTTTG-3’; shRNA2, 5’-CCGGCCTGATCACGTTTGACAACAACTCGAGTTGTTGTCAAACGTGATCAGGTTTTTG-3’; shRNA3, 5’-CCGGCGTCCTGATCACGTTTGACAACTCGAGTTGTCAAACGTGATCAGGACGTTTTTG-3’) were inserted into the pLKO.1 vector. Lentiviral particles were generated by co-transfecting the pLKO.1-shRNA plasmids with lentiviral-packing (psPAX2) and envelope (pMD2.G) plasmids into HEK293T cells. After transfection, lentiviral particles were harvested at 48 h and 72 h after transfection and subsequently added to HCC1954 cells. Following infection for 24 h, the cells were selected using 3 μg/ml puromycin for 3 days.

### CRISPR Knockout of TRPML1 in HCC1954 cells

CRISPR/Cas9 technique was used to knock out TRPML1 in HCC1954 cells. The targeted TRPML1 sequence, namely CTGCGACAAGTTTCGAGCCA AGG and TGCGACAAGTTTCGAGCCAA AGG (with AGG serving as the PAM motif) were targeted using the plentiCRISPR-V2 vector. Lentiviruses were generated by co-transfecting HEK293T cells with plentiCRISPR-V2, lentiviral-packing (psPAX2), and envelope (pMD2.G) plasmids via Lentifit (Hanbio) transfection reagent. After 48 h of transfection, the lentiviral particle-containing culture medium was harvested, filtered through a 0.45 μm membrane, and added to HCC1954 cells cultured in an antibiotic-free medium. The cells were subjected to puromycin selection at a concentration of 3 μg/mL starting from 24 h post-infection for a duration of 3 days. Single-cell clones were generated using a limiting dilution method and subsequently sequenced to validate TRPML1 knockout.

### Reverse transcription-quantitative polymerase chain reaction (RT-qPCR)

Total RNA was extracted from cells using the RNA isolater Total RNA Extraction Reagent (Vazyme) in accordance with the manufacturer’s instruction. The cDNA was obtained through reverse transcription using HiScript II Q RT SuperMix (Vazyme). RT-qPCR was conducted on LightCycler 96 (Roche) using AceQ qPCR SYBR Green Master Mix (Vazyme). The following primers were used: forward 5’-AACCAGCGCATGGACAGTTA-3’/reverse 5’-CGAGCTGGTCATGGAGTTGT-3’ for SOX2, forward 5’- GAGAACCGAGTGAGAGGCAAC-3’/reverse 5’-CTGATCTGCTGCAGTGTGGGT-3’ for OCT4, forward 5’- TTCTTCCACCAGTCCCAAAGG-3’/reverse 5’-TGCTGGAGGCTGAGGTATTT-3’ for NANOG, and forward 5’- TTCGCCGTCGTCTCAAATACT-3’/reverse 5’- CTCTTCCCGGAATGTCACAGC-3’ for TRPML1. mRNA relative level of target genes was normalized to TBP using forward 5’-TCCAGACTGGCAGCAAGAAAA-3’/reverse 5’-GAGCACAAGGCCTTCTAACCT-3’ primers. The relative mRNA levels of the target genes were calculated using the 2^-ΔΔCt^ method.

### ALDEFLUOR assay and FACS

The ALDH activity was measured using the ALDEFLUOR Kit (STEMCELL Technologies) following the manufacturer’s instructions. Briefly, 1 × 10^6^ HCC1954 cells or secondary mammospheres were isolated using trypsin and incubated with 5 μL of activated ALDEFLUOR reagent, BODIPY-aminoacetaldehyde (BAAA), in 1 mL ALDEFLUOR Assay Buffer. Immediately after mixing, 0.5 mL of the mixture was promptly added to the control tube containing 5 μL ALDEFLUOR DEAB reagent. Both samples were then incubated at 37 °C for 40 min before being centrifuged to remove supernatants and resuspended in 500 μL ALDEFLUOR Assay Buffer. Stained cells were analyzed using a CytoFLEX flow cytometer (Beckman Coulter) to determine the proportion of CSCs or sorted by a Moflo Astrios flow cytometer (Beckman Coulter) to isolate CSCs.

### Electrophysiology

Patch clamp recordings were performed using a Multiclamp 700B or Axopatch 200B patch clamp amplifier, a Digidata 1550B data acquisition system, and pClamp software (Molecular Devices). Whole-lysosome patch clamp recordings followed previously described method [23, 56]. Cells were pretreated with 1 μM vacuolin-1 overnight to enlarge endolysosomes. The bath solution contained 140 mM K-Gluconate, 4 mM NaCl, 1 mM EGTA, 2 mM MgCl_2_, 0.39 mM CaCl_2_, and 10 mM HEPES (pH 7.2). For whole-lysosome patch clamp recordings of HEK293T cells, the pipette solution contained 145 mM NaCl, 1 mM MgCl_2_, 5 mM KCl, 2 mM CaCl_2_, 10 mM MES, 10 mM HEPES and 10 mM Glucose (pH 4.6). In the experiment to verify the effect of Salinomycin on TRPML1-mediated iron flux, a pipette solution containing 105 mM ferrous gluconate, 10 mM HEPES, 1 mM HCl, 10 mM MES, and 90 mM glucose (pH 4.6) was employed. For whole-lysosome patch clamp recordings of HCC1954 cells or CSCs, the pipette solution contained 145 mM NaOH, 1 mM MgCl_2_, 5 mM KOH, 2 mM CaCl_2_, 10 mM MES, 10 mM Glucose, and 10 mM HEPES (pH 4.6, adjusted by methanesulfonic acid). The liquid junction potentials were corrected online. The inhibition curves presented in Fig. 1C were fitted with the equation

$$I/{I}_{0}=1/[1+(X/{{\rm{IC}}}_{50})h],$$

where *I* represents the current amplitude measured in the presence of the blocker, *I*_*0*_ represents the current amplitude measured in the absence of the blocker, *X* represents the concentration of the blocker, IC_50_ represents the concentration of the blocker that produces half-maximal inhibition, and *h* represents the Hill coefficient.

### Cell viability assay

Cell Counting Kit-8 (CCK-8) (TargetMol) was used to evaluate cell viability. Cells were seeded at a density of 8000 cells per well in 96-well microplates, followed by drug treatment as indicated. Subsequently, the culture medium was removed and replaced with fresh medium containing 10% (v/v) of the CCK-8 medium to assess the cell viability. The results were expressed as the ratio of absorbance at 450 nm to that of control cells. The test was performed in triplicate and repeated at least three times.

### Wound-healing assay

WT and TRPML1-KO HCC1954 cells were seeded into 6-well plates and cultured in complete medium until they reached approximately 90% confluence. Subsequently, the cells were scratched using a 1 mL pipette tip, washed twice with prewarmed PBS to eliminate any cellular debris, and cultured in medium containing 2% FBS. Wound healing was observed under a microscope (Olympus, IX 73) at both time points of 0 h and 48 h. Migration rate was calculated as (D_0_ − D_t_)/D_0_, where D_0_ represents the initial distance between the wounds at 0 h, and D_t_ represents the distance between the wounds at 48 h.

### CFSE cell proliferation assay

To assess cell proliferation, we utilized the CellTrace CFSE Cell Proliferation Kit (Thermo Fisher Scientific). A total of 10^5^ WT or TRPML1-knockout HCC1954 cells were stained with 5 μM CFSE dye for 20 min at 37 °C, followed by the addition of RPMI 1640 complete medium supplemented with 10% FBS. Subsequently, the cells were washed twice with warm PBS and seeded into 6-well plates. After incubating for 48 h at 37 °C, the cells were harvested and washed twice with ice-cold PBS. The fluorescence emitted upon excitation by a laser at a wavelength of 488 nm was quantified using flow cytometry to determine CFSE fluorescence intensity.

### Mammosphere formation

HCC1954 cancer cells were cultured with MammoCult Basal Medium (StemCell) containing 10% Heparin (StemCell), Hydrocortisone (Aladdin), and 1% penicillin-streptomycin (Biosharp). The cells were seeded at a density of 1 × 10^5^ cells/ml into 100-mm dishes coated with 1% agar at the bottom and cultured for 7 days. Subsequently, the mammosphere was collected and trypsinized, and the resulting cells were plated into 6-well plate with ultra-low attachment surface (Corning) to form secondary mammospheres. On the following day, the secondary mammospheres were treated with either DMSO as a control or specific drugs as indicated. The mammospheres were then cultured for an additional 2 days before undergoing ALDEFLUOR assay and ROS/lipid ROS detection, or for another 7 days before being visualized using an inverted microscope and quantified based on the number of mammospheres with a diameter greater than or equal to 50 μm.

### Measurement of ROS production

Reactive oxygen species (ROS) levels were measured by flow cytometry using CM-H2DCFDA (Invitrogen). Briefly, HCC1954 cells or secondary mammospheres (mentioned above) were treated with DMSO as a control or 10 μM ML-SI1 for 48 h. Subsequently, the cells were trypsinized and incubated with 5 μM CM-H2DCFDA at 37 °C for 40 min. Afterward, the cells were washed twice with ice-cold PBS and counterstained with DAPI (1 μg/mL) to exclude dead cells. The ROS and DAPI signals were analyzed on the 525/40 BP and 450/45 BP channels of a CytoFLEX cytometer (Beckman Coulter), respectively. The mean fluorescence intensity was used to indicate the ROS level.

### Lipid ROS detection

Lipid ROS levels were measured using flow cytometry or fluorescence microscopy with BODIPY 581/591 C11 (Thermo Fisher Scientific). For fluorescence microscopy analysis, the secondary mammospheres (mentioned above) were treated with DMSO or ML-SI1 (10 μM) for 48 h. Subsequently, the cells were trypsinized and plated into poly-L-lysine-coated chambers (NEST) for 1 h, followed by incubation in growth medium containing 2 μM BODIPY 581/591 C11 at 37 °C for 1 h. Afterward, the cells were rinsed twice with ice-cold PBS prior to being visualized using spinning-disc confocal microscopy. The fluorescence was excited by a 488 nm laser and detected through a 525/50 nm filter (Semrock). For flow cytometry analysis, cells were incubated with 2 μM BODIPY 581/591 C11 for 1 h at 37 °C, followed by dispersion through trypsinization. After washed twice with PBS, the cells were counterstained with DAPI (1 μg/mL) to exclude non-viable cells. The oxidation of BODIPY 581/591 C11 led to a shift in the fluorescence emission peak from 590 nm to 510 nm, which was indicative of lipid ROS generation and subsequently analyzed using a CytoFLEX cytometer.

### Malondialdehyde (MDA) and reduced glutathione (GSH) assay

HCC1954 cells were treated with 10 μM ML-SI1 for 48 h in the presence or absence of either 1 μM Liproxstatin-1 (Lip-1) or 20 μM deferoxamine (DFO). Subsequently, the cells were collected in ice-cold PBS and lysed using sonication for 30 s. The resulting lysate was then centrifuged at 12,000 × *g* for 10 minutes at 4 °C, and the supernatant was collected. The protein concentration was determined using BCA protein assay (Beyotime). The levels of MDA and GSH were assessed with the commercial kits for MDA and GSH (Nanjing Jiancheng Bioengineering Institute) according to the manufacturer’s instructions on a Spark multimode microplate reader (TECAN).

### Determination of intracellular Fe^2+^

Intracellular Fe^2+^ accumulation was assessed using the FeRhoNox-1 fluorescent probe (Maokang Biotechnology). HCC1954 cells were treated with DMSO or 10 μM ML-SI1 for 48 h. Subsequently, the cells were incubated at 37 °C for additional 30 min in culture medium containing 5 μM FeRhoNox-1 and 1 μM LysoTracker Green (Thermo Fisher Scientific). Prior to imaging, the cells were rinsed twice with warm-PBS. All images were captured using an Olympus SpinSR confocal microscope, employing lasers with wavelengths of 561 nm and 488 nm to excite FeRhoNox-1 and lysotracker green respectively.

### Western blotting

Cells were lysed in ice-cold lysis buffer containing 1% NP-40, 50 mM Tris HCl, 150 mM NaCl, 5 mM EDTA, and a working concentration of the protease inhibitor (Thermo Fisher Scientific) and incubated on ice for 30 min with a gentle shake. The lysate was then centrifuged at 18,506 × *g* for 10 min at 4 °C. 20 μl of the supernatant was collected and incubated with BCA reagent (Beyotime) at 37 °C for 30 min to determine the protein concentration. After quantification, the cell lysates were heated in a sample buffer at 72 °C for 15 min. Then the lysates were loaded and subjected to 10% sodium dodecyl sulfate–polyacrylamide gel electrophoresis (SDS–PAGE), and electrophoretically transferred onto Immobilon-P polyvinylidene difluoride (PVDF) membranes (Millipore) using a vertical electrophoresis system and a Trans-Blot electrophoretic transfer system (Cavoy). The membranes were blocked with 5% non-fat dry milk in Tris-buffered saline containing 0.1% Tween 20 (TBST) for 2 h at room temperature and incubated with primary antibodies against TRPML1 (Abcam, ab28508, 1:1000; Santa Cruz Biotechnology, sc-398868, 1:100), Ferritin (proteintech, 10727-1-AP, 1:5000), and ACTB (proteintech, 20536-1-AP, 1:5000) in 5% non-fat dry milk in TBST overnight at 4 °C. The blots were washed three times for 10 min each in TBST and then incubated with HRP-labeled secondary antibodies (proteintech, SA00001, 1:2000) for 2 h at room temperature. After the reaction, the blots were washed three times for 10 min in TBST, and then incubated with Western Bright Sirius HRP substrate (Advansta, K-12043-D20) for 2 min and detected by a gel imaging system (ChemiDocIt510, UVP). Full and uncropped images for western blots can be found in the Supplemental Materials.

### Xenograft tumor formation

WT and TRPML1-knockout HCC1954 cells were isolated using trypsin and cultured under mammosphere culture conditions as mentioned above. All the animals were randomly divided into experimental and control groups. On day 7, the mammospheres were collected. Subsequently, the secondary mammosphere was treated with either vehicle (DMSO) or 20 μM ML-SI1 (TargetMol). After 3 days, the mammospheres were collected and enzymatically digested. The cells were then washed with PBS and resuspended in a mixture of PBS and Matrigel at a 1:1 volume ratio. 125 μL of cell suspension was then injected into the second pair of mammary gland fat pads of 4~6-week-old female BALB/c nude mice. Tumor sizes were measured every other day (Fig. 7A–C) or twice a week (Fig. 7D, E and Supplementary Fig. S4) with calipers, and calculated as tumor volume = Length × Width^2^/2.

### Data analysis

Sample sizes are chosen based on statistical analysis and standard in the field. The data were analyzed using Clampfit (Molecular Devices), OriginPro (OriginLab) and Excel (Microsoft). The test was repeated at least three times. Numeric data are shown as the mean ± SEM. Statistical significance was calculated with one-way analysis of variance (ANOVA) or Student’s *t* tests and is indicated with * (* for *p* < 0.05, ** for *p* < 0.01, and *** for *p* < 0.001).

### Supplementary information

Supplementary Figures

Original images of western blots

## Data Availability

All data needed to evaluate the conclusions that are present in the paper or the Supplementary information. The data generated in this study are available within the article and its Supplementary Data files.
