# Supplementary material for: Inhibition of lysosomal TRPML1 channel eliminates breast cancer stem cells by triggering ferroptosis
Source: Cell Death Discov. 2024 May 27;10:256. doi: 10.1038/s41420-024-02026-y (PMC11130215; doi:10.1038/s41420-024-02026-y)
Supplement: Supplementary file 1 — Supplementary Figures [file 41420_2024_2026_MOESM1_ESM.pdf]

Supplementary Figures

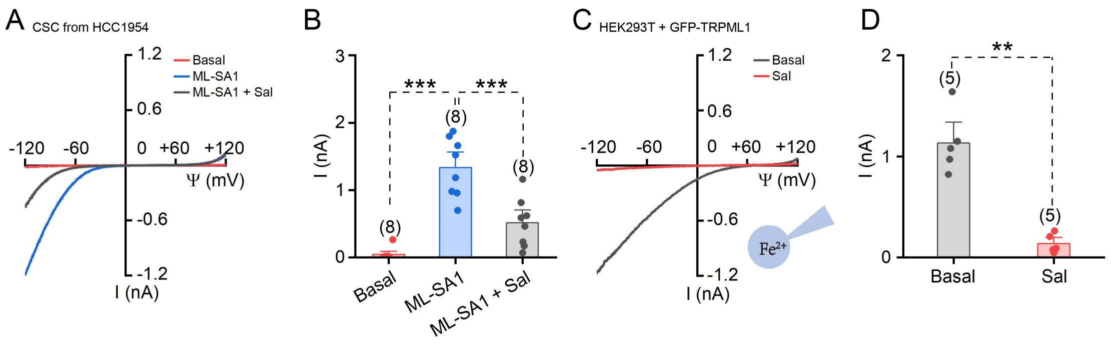

Supplementary Fig. S1. Salinomycin blocks lysosomal cation channel TRPML1 in CSCs and HEK293T cells.

(**A**) Representative TRPML1 lysosomal currents recorded in HCC1954 CSCs with 10 μM ML-SA1 alone or a combination of 10 μM ML-SA1 and 5 μM salinomycin (Sal) applied in bath solution. (**B**) Current amplitudes measured at −120 mV in recordings shown in (A). (**C**) Representative TRPML1 lysosomal currents recorded in GFP-TRPML1 transfected HEK293T cells with 5 μM Sal bath application. The pipette (lumen) solution contained 105 mM Fe^{2+} (pH 4.6). (**D**) Current amplitudes measured at −120 mV in recordings shown in (C). Data are presented as the means ± SEM.

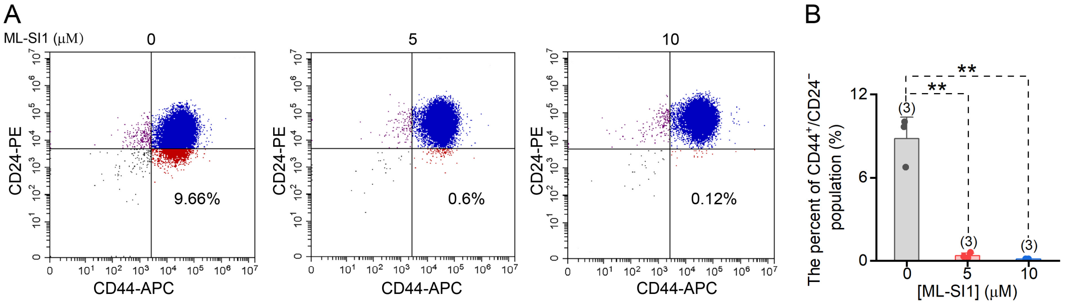

Supplementary Fig. S2. Inhibition of TRPML1 reduces the CD44$^+$/CD24$^-$ fraction in HCC1954 cells.

(**A**) Representative flow cytometry analysis of CD44$^+$/CD24$^-$ fraction in HCC1954 cells following 48 h treatment of different concentrations of ML-SI1. (**B**) Quantification of the CD44$^+$/CD24$^-$ fraction shown in (A). Data are presented as the means ± SEM.

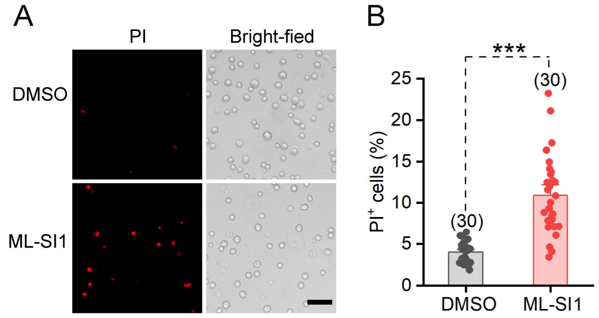

Supplementary Fig. S3. Treatment with 20 µM ML-SI1 induces cell death in HCC1954 cells.

(**A**) HCC1954 cells were treated with 20 µM ML-SI1 for 48 h and then stained with propidium iodide (PI) to visualize the dead cells. Scale bar = 200 µm. (**B**) Quantitative analysis of the PI^+ cells shown in (A). Data are presented as the means ± SEM.

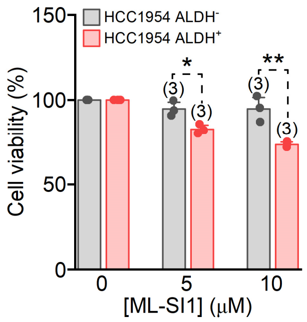

Supplementary Fig. S4. CSCs exhibit greater sensitivity to ML-SI1 compared to non-CSCs.

CSCs and non-CSCs were isolated from HCC1954 cells. The cell viability was evaluated using the CCK8 assay. The percentage of viable cells in relation to DMSO treatment, following exposure to ML-SI1 for 48 h, is indicated.

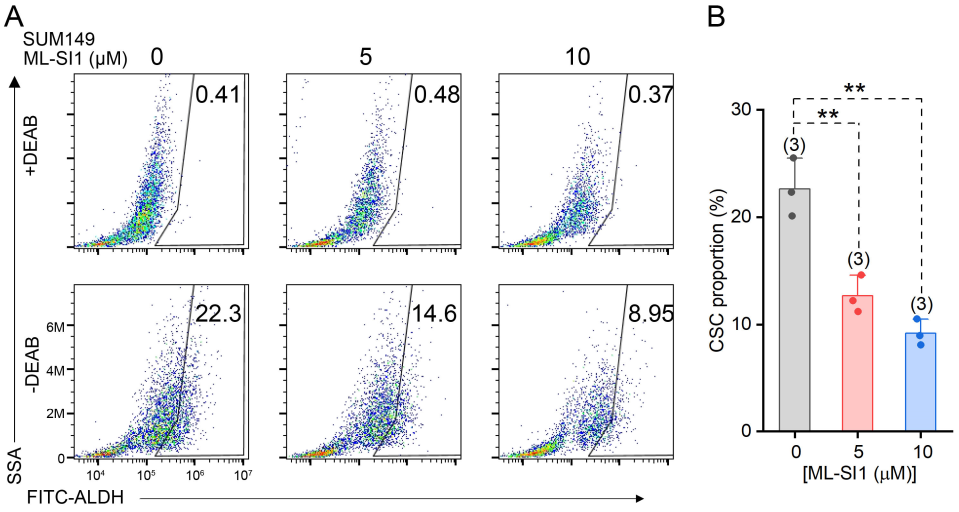

Supplementary Fig. S5. Inhibition of TRPML1 reduces the proportion of CSC in SUM149 mammospheres.

(A) Representative flow cytometry analysis of CSC proportion in SUM149 mammospheres following 48 h treatment of different concentrations of ML-SI1. **(B)** Quantification of CSC proportions in SUM149 mammospheres as shown in (A).

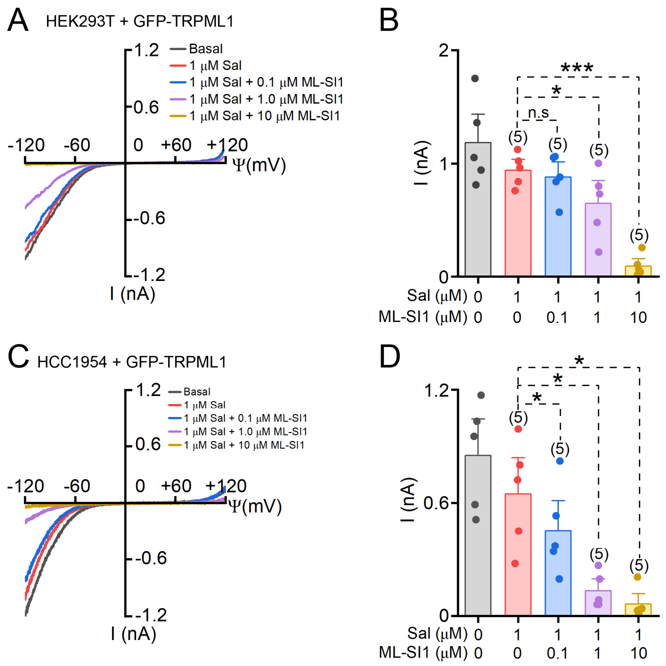

Supplementary Fig. S6. Combination of salinomycin and ML-SI1 effectively inhibits lysosomal TRPML1 current.

(**A**) Representative whole-lysosomal TRPML1 currents recorded in GFP-TRPML1 transfected HEK293T cells after bath application of 1 µM salinomycin and different concentrations of ML-SI1 as indicated.

(**B**) Statistics of the current amplitudes measured at −120 mV in (A).

(**C**) Representative whole-lysosomal TRPML1 currents recorded in GFP-TRPML1 transfected HCC1954 cells after bath application of 1 µM salinomycin and different concentrations of ML-SI1 as indicated.

(**D**) Statistics of the current amplitudes measured at −120 mV in (C).

The currents were recorded with ramp voltage protocols (−120 to + 120 mV in 2 s, Vh = 0 mV).

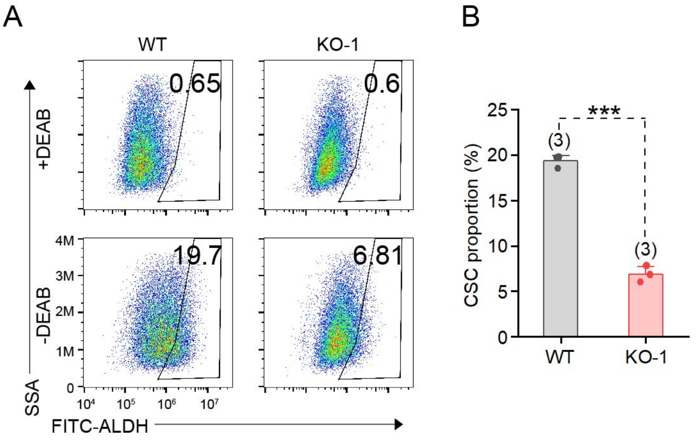

Supplementary Fig. S7. Knockout of TRPML1 reduces the proportion of CSCs in mammospheres.

(**A**) The proportions of CSCs in WT and TRPML1-KO mammosphere cells assessed using the ALDEFLUOR assay and analyzed by FACS. (**B**) Statistics of the CSC proportion in (A). Data are presented as the means ± SEM.

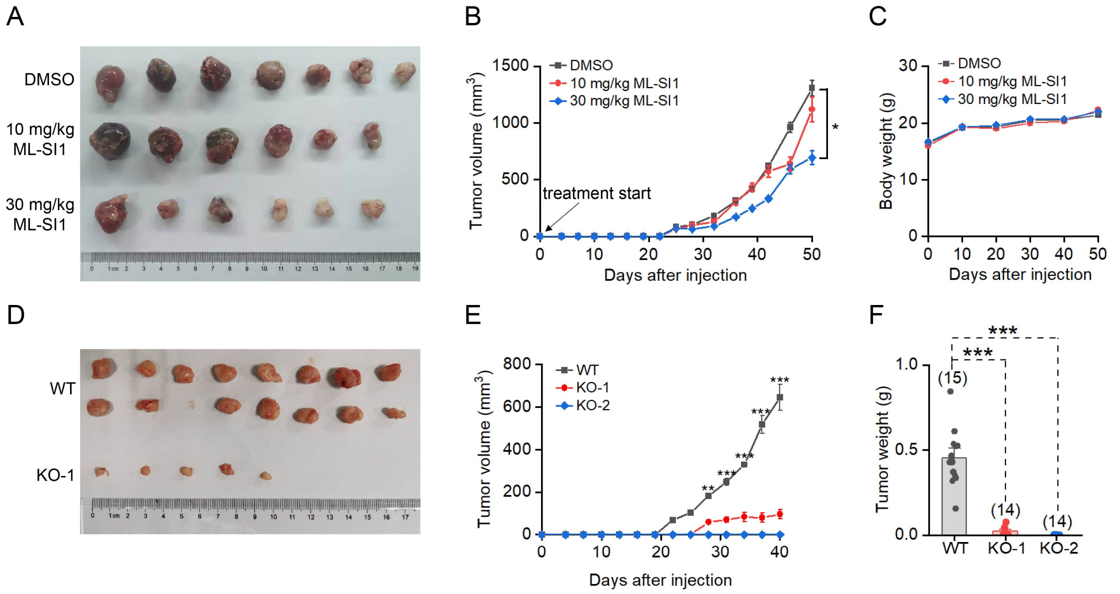

Supplementary Fig. S8. Tumor volume and tumor weight in the breast cancer xenografts.

(**A**) Tumors isolated from xenograft model mice that received intraperitoneal injections of either vehicle (DMSO) or varying doses of ML-SI1 every other day. Following a 50-day treatment period, the tumors were excised and photographed. (**B**) Growth curves of the tumors shown in (A). Tumor sizes were monitored by measuring the perpendicular diameter with calipers twice a week, and calculated with the following equation: tumor volume = length × width^2 / 2. (**C**) Body weights of the mice shown in (A). (**D**) Tumors isolated from xenograft model mice established by orthotopic injection of dispersed WT or TRPML1-KO HCC1954 cells into the mammary fat pads of nude mice. (**E**) Growth curves of the tumors shown in (D). (**F**) Weights of the tumors shown in (D).
