# Supplementary material for: Inhibition of lysosomal TRPML1 channel eliminates breast cancer stem cells by triggering ferroptosis
Source: Cell Death Discov. 2024 May 27;10:256. doi: 10.1038/s41420-024-02026-y (PMC11130215; doi:10.1038/s41420-024-02026-y)

Original images of western blots

Fig. 1D

TRPML1

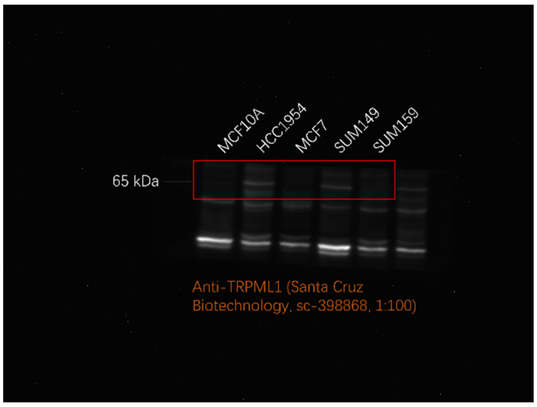

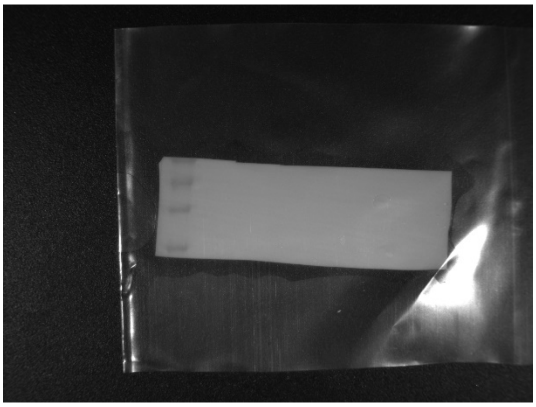

Actin

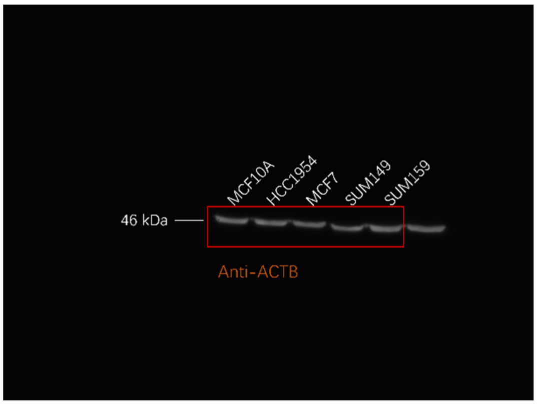

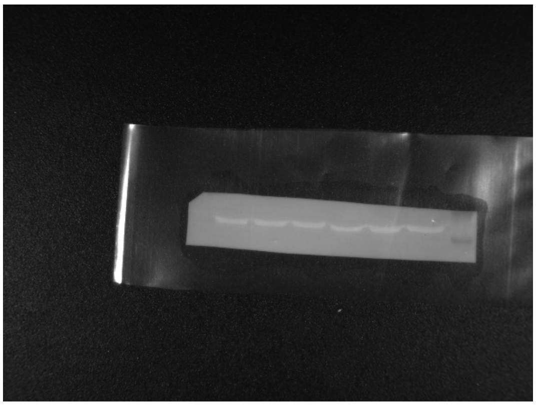

Fig. 3E

Ferritin

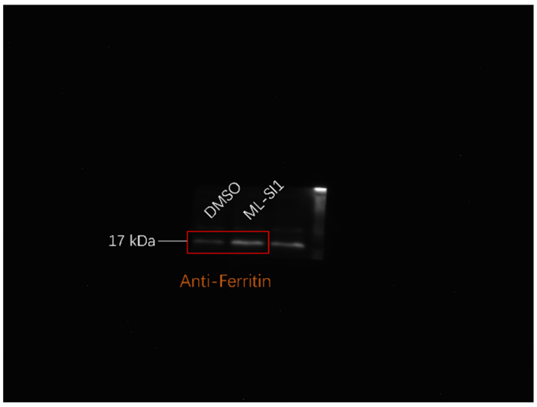

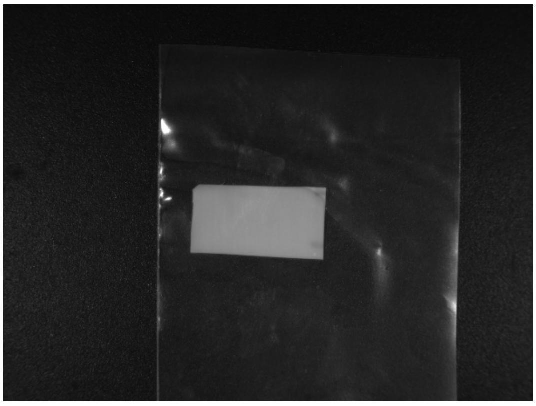

Actin

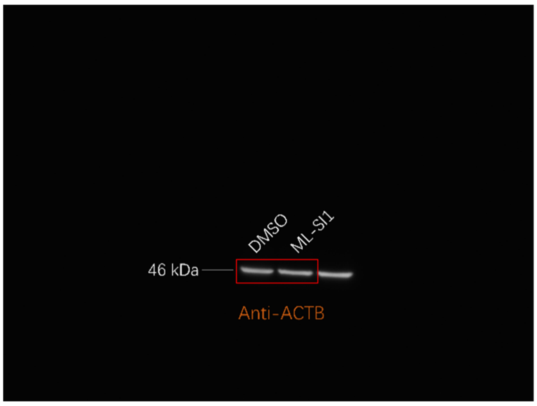

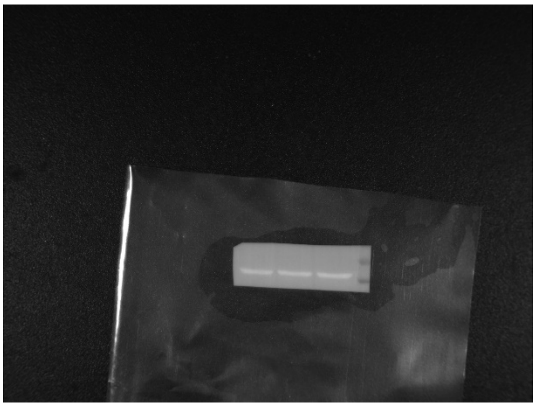

Fig. 5D

TRPML1

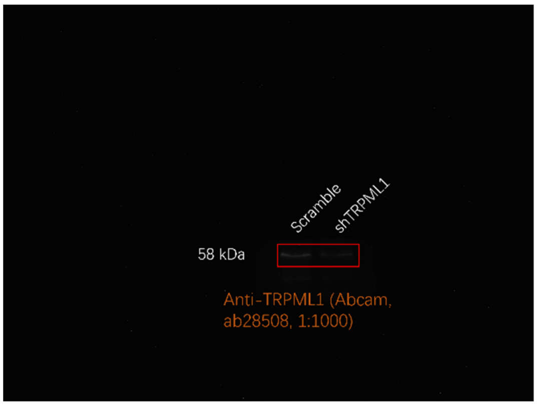

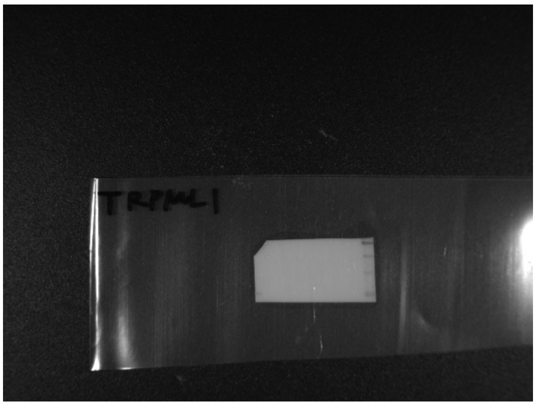

Actin

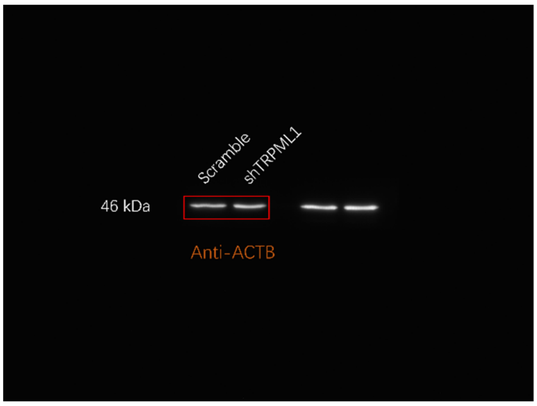

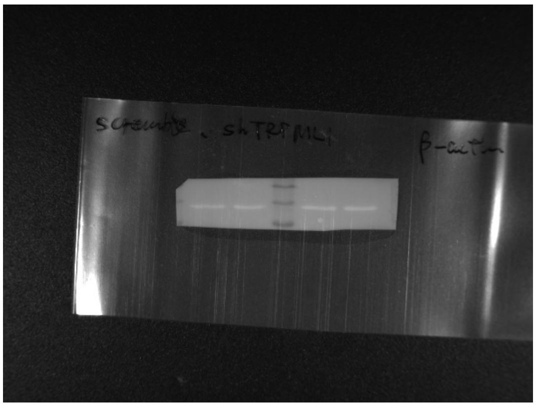

Fig. 6B

TRPML1

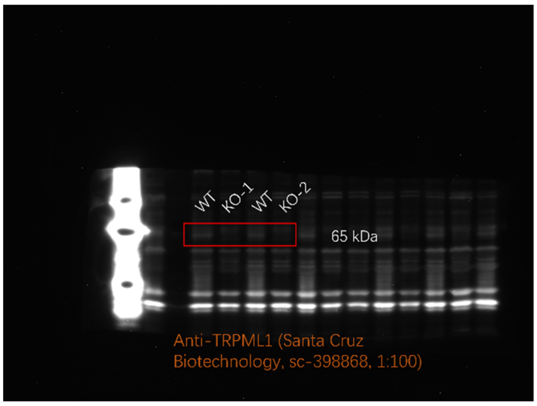

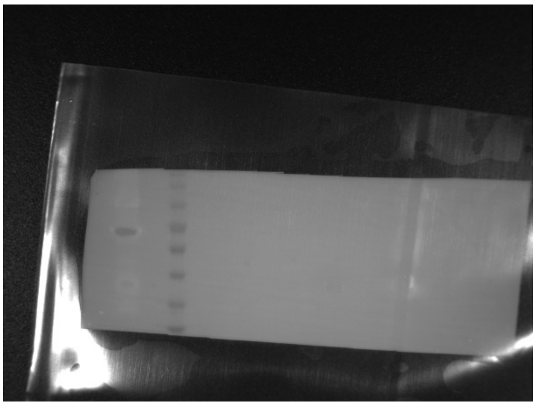

Actin

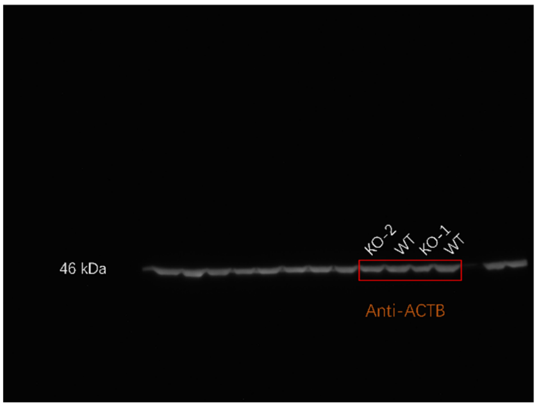

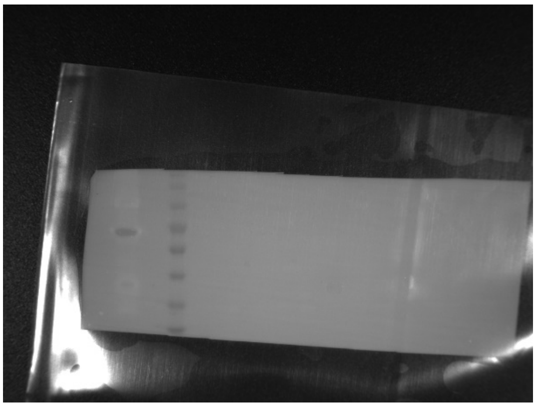

Fig. 6H

Ferritin

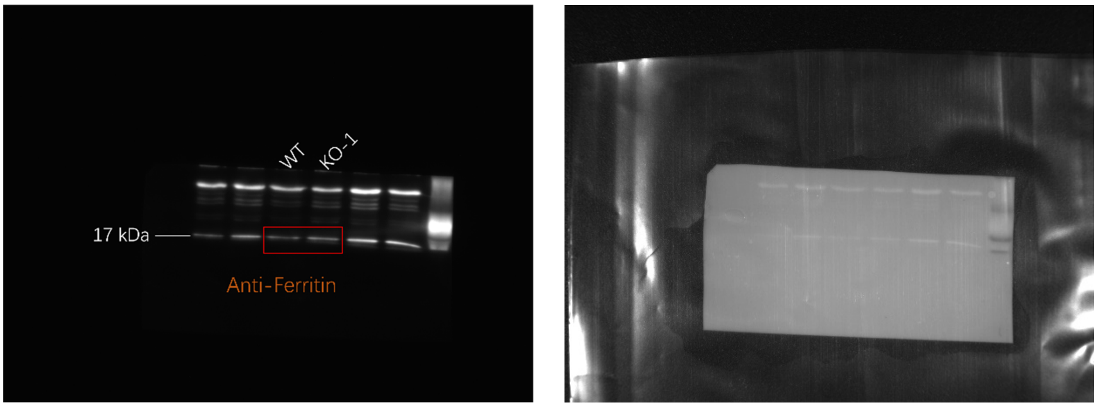

Actin

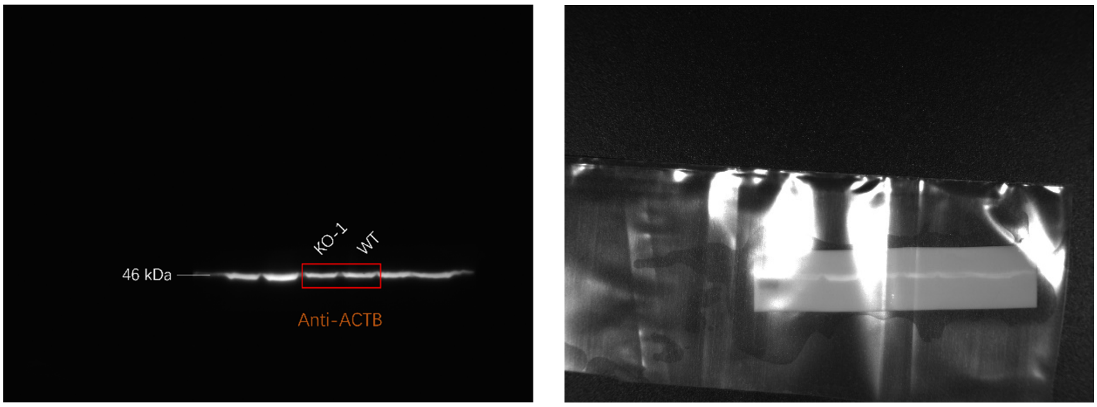

Supplement: Supplementary file 2 — Original images of western blots [file 41420_2024_2026_MOESM2_ESM.pdf]
